# Prevalence and outcomes of atrial fibrillation in patients suffering prostate cancer: a national analysis in the United States

**DOI:** 10.3389/fcvm.2024.1382166

**Published:** 2024-04-04

**Authors:** Zhemin Pan, Xiao Xu, Xi Xu, Shengyong Wu, Zhensheng Zhang, Suxuan Liu, Zhijian Liu, Boxiang Tu, Chenxin Chen, Yingyi Qin, Jia He

**Affiliations:** ^1^ Tongji University School of Medicine, Shanghai, China; ^2^ Department of Urology, Changhai Hospital, Naval Medical University, Shanghai, China; ^3^ Department of Military Health Statistics, Naval Medical University, Shanghai, China; ^4^ Department of Cardiology, Changhai Hospital, Naval Medical University, Shanghai, China; ^5^ Department of Nursing, The 940 Hospital of Joint Logistic Support Force of PLA, Lanzhou, China

**Keywords:** atrial fibrillation, clinical outcomes, prevalence, prostate cancer, risk predicator

## Abstract

**Purpose:**

Although the adverse effects of atrial fibrillation (AF) on cancers have been well reported, the relationship between the AF and the adverse outcomes in prostate cancer (PC) remains inconclusive. This study aimed to explore the prevalence of AF and evaluate the relationship between AF and clinical outcomes in PC patients.

**Methods:**

Patients diagnosed with PC between 2008 and 2017 were identified from the National Inpatient Sample database. The trends in AF prevalence were compared among PC patients and their subgroups. Multivariable regression models were used to assess the associations between AF and in-hospital mortality, length of hospital stay, total cost, and other clinical outcomes.

**Results:**

256,239 PC hospitalizations were identified; 41,356 (83.8%) had no AF and 214,883 (16.2%) had AF. AF prevalence increased from 14.0% in 2008 to 20.1% in 2017 (*P* < .001). In-hospital mortality in PC inpatients with AF increased from 5.1% in 2008 to 8.1% in 2017 (*P* < .001). AF was associated with adverse clinical outcomes, such as in-hospital mortality, congestive heart failure, pulmonary circulation disorders, renal failure, fluid and electrolyte disorders, cardiogenic shock, higher total cost, and longer length of hospital stay.

**Conclusions:**

The prevalence of AF among inpatients with PC increased from 2008 to 2017. AF was associated with poor prognosis and higher health resource utilization. Better management strategies for patients with comorbid PC and AF, particularly in older individuals, are required.

## Introduction

Atrial fibrillation (AF) is the most common arrhythmia seen clinically. The incidence of AF is increasing due to the aging population, especially in patients who have suffered from cancer (1). The incidence of AF has been increasing every year in recent years, and this trend has been exacerbated by the increasing lives expectancy of the cancer patient and the use of various cancer-related treatments. Research confirms that the pro-inflammatory status in cancer predispose to the development of arrhythmia (2, 3). Novel chemotherapeutic agents, radiation therapy, and improved cancer-related survival have all increased the importance of other concomitant medical conditions in cancer patients, particularly cardiovascular diseases (CVD) (4).

The study of the association between cancers and cardiovascular diseases is referred to as onco-cardiology (5). There are numerous articles indicating the close links between cancer and AF (6, 7). Prostate cancer (PC) is the most common visceral tumor and the second most common cause of cancer-related death in males (8). Studies have shown that in all age groups, mortality, hospitalization costs, and length of hospital stay are higher in cancer patients with AF compared to those without AF (9). In patients over 80 years of age, AF has a significant association with PC, which dramatically worsens the mortality of patients with PC (9). ADT, Androgen Deprivation Treatment, a widely used chemical treatment for prostate cancer, is associated with QT prolongation (LQT) and Torsade de Pointes (TdP) through blockade of testosterone effects on ventricular repolarization (10).

However, there are limited reports regarding the impact of AF on patients with PC. We therefore carry out this large scale, population-based study to determine the association of AF with PC and its subsequent impact on in-hospital outcomes.

## Materials and methods

### Data source

This study used inpatient data obtained from the National Inpatient Sample Database (NIS) database between 2008 and 2017. The NIS is the largest publicly available all-payer data base established by the Agency for Healthcare Research and Quality under the Healthcare Cost and Utilization Project. More details regarding the NIS are available at www.hcup-us.ahrq.gov. Our study was exempt from formal Institutional Review Board approval owing to the use of publicly available database.

### Study population

**Figure 1:**
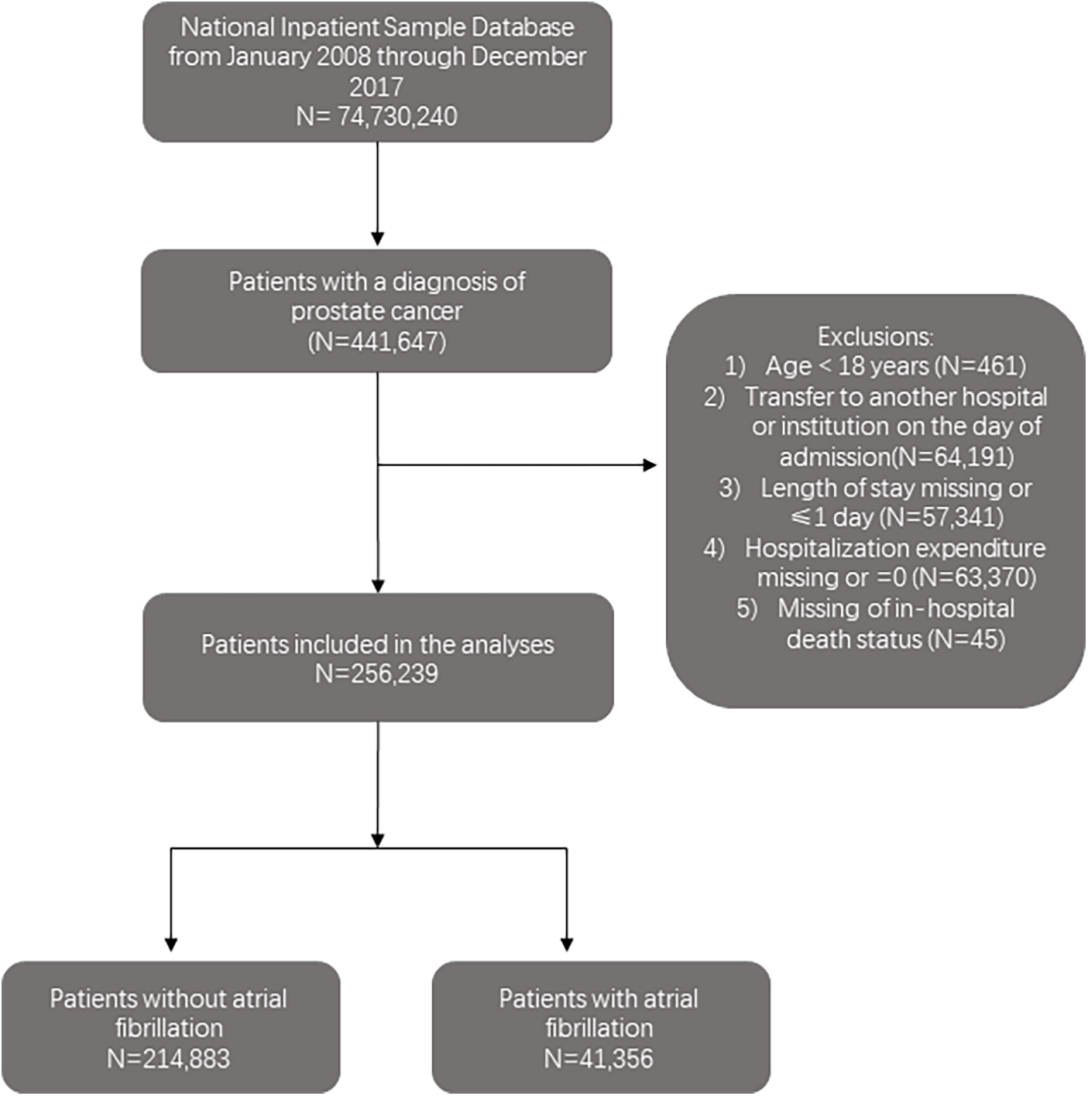
The flowchart of the cohort selection process.

Of 74,730,240 hospitalizations recorded between 2008 and 2017 in the NIS database, 256,239 hospitalization records with patients aged ≥18 years old with a prostate cancer (PC) diagnosis were included. International Classification of Disease, Ninth Revision (ICD-9) and Tenth Revision (ICD-10) codes were used to identify PC hospitalizations (ICD-9-CM codes including 233.4 or 185 and ICD-10-CM codes D07.5 or C61) (11). Records that meet the following criteria were excluded: (1) age <18 years; (2) transferred to another hospital or institution on the day of admission; (3) length of stay missing or ≤1 day; (4) hospitalization expenditure missing or was 0 and (5) missing in-hospital death status. The study cohort was divided into two groups based on the presence or absence of atrial fibrillation (AF) (ICD-9-CM code 427.3 and ICD-10-CM code I48). Figure 1 illustrates the steps used to locate the target population.

### Covariate assessment

Information regarding patient- and hospital-level characteristics for each hospitalization is included in the NIS. The patient-level variables were age, sex, race, median household income of residents in the patient' s ZIP Code, insurance type, admission day and admission season. Hospital-level characteristics included the region, teaching status, bed size, and hospital ownership. We also collected information on comorbidities, as suggested in previous studies, such as alcohol abuse, drug misuse, coronary heart disease, lipoid metabolism disorders, atherosclerosis, nephrotic syndrome, thyrotoxicosis, and chronic kidney disease, according to ICD-9/10 diagnostic codes and procedure codes (12). All the variables are shown in the first column of Table 1. Corresponding ICD codes of these variables are listed in Supplementary Table S1.

**Table 1:** Characteristics of records with and without AF in PC patients.

Variables	Overall	Without Atrial Fibrillation	With Atrial Fibrillation	*P*-value
	1,275,345.4	1,069,372.5 (83.8)	205,972.9 (16.2)	
Age group (%)				<0.001
18–44	4,938.5 (0.4)	4,901.9 (0.5)	36.6 (0.0)	
45–64	344,542.7 (27.0)	329,355.6 (30.8)	15,187.1 (7.4)	
65–74	388,159.6 (30.4)	339,351.4 (31.7)	48,808.2 (23.7)	
≥75	537,704.7 (42.2)	395,763.5 (37.0)	141,941.1 (68.9)	
Race (%)				<0.001
White	807,363.9 (63.3)	654,316.3 (61.2)	153,047.6 (74.3)	
Black	206,362.2 (16.2)	186,792.3 (17.5)	19,570.0 (9.5)	
Hispanic	81,185.2 (6.4)	72,054.5 (6.7)	9,130.7 (4.4)	
Asian or Pacific Islander	21,084.4 (1.7)	18,143.9 (1.7)	2,940.5 (1.4)	
Other	37,595.8 (2.9)	33,407.7 (3.1)	4,188.1 (2.0)	
Missing	121,753.9 (9.5)	104,657.8 (9.8)	17,096.1 (8.3)	
Male (%)	1,275,345.4 (100.0)	1,069,372.5 (100.0)	205,972.9 (100.0)	NA
Admission season (%)				0.492
Winter	311,553.4 (24.4)	261,150.9 (24.4)	50,402.5 (24.5)	
Spring	366,123.8 (28.7)	307,157.2 (28.7)	58,966.6 (28.6)	
Summer	300,768.3 (23.6)	252,631.0 (23.6)	48,137.3 (23.4)	
Autumn	296,899.9 (23.3)	248,433.4 (23.2)	48,466.6 (23.5)	
Insurance type (%)				<0.001
Medicare	838,109.0 (65.7)	664,174.1 (62.1)	173,934.9 (84.4)	
Medicaid	53,380.6 (4.2)	49,738.3 (4.7)	3,642.3 (1.8)	
Private insurance	331,118.5 (26.0)	307,582.4 (28.8)	23,536.0 (11.4)	
Self-pay	17,861.7 (1.4)	16,664.3 (1.6)	1,197.5 (0.6)	
Other	34,875.6 (2.7)	31,213.4 (2.9)	3,662.2 (1.8)	
Elective admission (%)	470,908.3 (36.9)	432,471.8 (40.4)	38,436.5 (18.7)	<0.001
Income quartile (%)				<0.001
0–25th	362,762.4 (28.4)	312,375.7 (29.2)	50,386.6 (24.5)	
26–50th	312,805.8 (24.5)	261,058.4 (24.4)	51,747.5 (25.1)	
51–75th	303,403.0 (23.8)	252,874.5 (23.6)	50,528.5 (24.5)	
76–100th	296,374.3 (23.2)	243,063.9 (22.7)	53,310.3 (25.9)	
Control/ownership of hospital (%)				<0.001
Government, nonfederal	149,353.1 (11.7)	129,157.2 (12.1)	20,195.9 (9.8)	
Private, not-profit	976,800.9 (76.6)	814,139.6 (76.1)	162,661.3 (79.0)	
Private, invest-own	149,191.4 (11.7)	126,075.7 (11.8)	23,115.7 (11.2)	
Hospital bedsize (%)				<0.001
Small	175,872.9 (13.8)	145,207.3 (13.6)	30,665.6 (14.9)	
Medium	318,153.4 (24.9)	265,522.1 (24.8)	52,631.3 (25.6)	
Large	781,319.1 (61.3)	658,643.1 (61.6)	122,676.0 (59.6)	
Location/teaching status of hospital (%)				<0.001
Rural	129,724.1 (10.2)	107,124.1 (10.0)	22,600.1 (11.0)	
Urban nonteaching	426,335.5 (33.4)	351,954.9 (32.9)	74,380.6 (36.1)	
Urban teaching	719,285.8 (56.4)	610,293.6 (57.1)	108,992.2 (52.9)	
Region of hospital (%)				<0.001
Northeast	272,045.8 (21.3)	225,173.3 (21.1)	46,872.5 (22.8)	
Midwest	310,314.5 (24.3)	258,937.3 (24.2)	51,377.2 (24.9)	
South	460,438.5 (36.1)	391,819.3 (36.6)	68,619.2 (33.3)	
West	232,546.7 (18.2)	193,442.6 (18.1)	39,104.1 (19.0)	
Mean CHA2DS2-VASc score (SD)	2.42 (1.60)	2.24 (1.56)	3.33 (1.48)	<0.001
Clinical Outcomes and complications				
Death (%)	54,195.9 (4.2)	38,433.6 (3.6)	15,762.4 (7.7)	<0.001
Congestive heart failure (%)	118,410.1 (9.3)	72,649.5 (6.8)	45,760.7 (22.2)	<0.001
Pulmonary circulation disorders (%)	23,261.9 (1.8)	15,957.8 (1.5)	7,304.0 (3.5)	<0.001
Renal failure (%)	203,038.2 (15.9)	149,133.5 (13.9)	53,904.7 (26.2)	<0.001
Fluid and electrolyte disorders (%)	321,116.6 (25.2)	254,008.9 (23.8)	67,107.7 (32.6)	<0.001
Cardiogenic shock (%)	3,350.3 (0.3)	1,885.8 (0.2)	1,464.4 (0.7)	<0.001
Median total cost [IQR]	10,977.06 [6,706.20, 17,369.59]	11,045.36 [6,784.84, 17,179.64]	10,563.17 [6,335.66, 18,596.05]	0.489
Median LOS [IQR]	3.00 [2.00, 6.00]	3.00 [2.00, 6.00]	4.00 [3.00, 7.00]	<0.001
Lymphatic metastasis (%)	19,160.7 (1.5)	17,500.5 (1.6)	1,660.2 (0.8)	<0.001
Brain metastases (%)	14,609.6 (1.1)	12,949.0 (1.2)	1,660.6 (0.8)	<0.001
Osseous metastasis (%)	263,076.7 (20.6)	217,993.2 (20.4)	45,083.5 (21.9)	<0.001
Pulmonary metastasis (%)	32,859.1 (2.6)	27,613.4 (2.6)	5,245.6 (2.5)	0.677
Hepatic metastases (%)	41,480.3 (3.3)	35,432.9 (3.3)	6,047.4 (2.9)	<0.001
Smoking (%)	346,676.3 (27.2)	290,047.1 (27.1)	56,629.1 (27.5)	0.122
Coronary heart disease (%)	349,651.5 (27.4)	254,753.5 (23.8)	94,898.0 (46.1)	<0.001
Disorders of lipoid metabolism (%)	456,903.8 (35.8)	369,530.9 (34.6)	87,372.9 (42.4)	<0.001
Atherosclerosis (%)	325,888.0 (25.6)	238,358.5 (22.3)	87,529.6 (42.5)	<0.001
Nephrotic syndrome (%)	973.1 (0.1)	832.8 (0.1)	140.3 (0.1)	0.512
Thyrotoxicosis (%)	2,297.6 (0.2)	1,615.6 (0.2)	681.9 (0.3)	<0.001
Chronic kidney disease (%)	230,531.9 (18.1)	170,971.6 (16.0)	59,560.3 (28.9)	<0.001
Injection or infusion of cancer chemotherapeutic substance (%)	10,474.9 (0.8)	9,515.1 (0.9)	959.7 (0.5)	<0.001
Therapeutic radiology and nuclear medicine (%)	40,333.9 (3.2)	34,930.7 (3.3)	5,403.2 (2.6)	<0.001
Alcohol abuse (%)	31,610.7 (2.5)	26,725.8 (2.5)	4,884.9 (2.4)	0.128
Rheumatoid arthritis/collagen vascular diseases (%)	17,466.7 (1.4)	13,638.2 (1.3)	3,828.5 (1.9)	<0.001
Deficiency anemias (%)	297,824.9 (23.4)	237,898.1 (22.2)	59,926.8 (29.1)	<0.001
Acquired immune deficiency syndrome (%)	1,655.1 (0.1)	1,520.5 (0.1)	134.6 (0.1)	<0.001
Chronic blood loss anemia (%)	22,923.1 (1.8)	18,370.3 (1.7)	4,552.8 (2.2)	<0.001
Chronic pulmonary disease (%)	211,647.9 (16.6)	162,629.0 (15.2)	49,018.9 (23.8)	<0.001
Coagulopathy (%)	79,198.8 (6.2)	60,033.3 (5.6)	19,165.5 (9.3)	<0.001
Diabetes uncomplicated (%)	248,362.4 (19.5)	204,222.1 (19.1)	44,140.3 (21.4)	<0.001
Diabetes with chronic complications (%)	59,401.3 (4.7)	45,669.9 (4.3)	13,731.4 (6.7)	<0.001
Depression (%)	85,529.1 (6.7)	71,369.8 (6.7)	14,159.3 (6.9)	0.137
Drug abuse (%)	12,350.8 (1.0)	11,238.3 (1.1)	1,112.5 (0.5)	<0.001
Hypertension (combine uncomplicated and complicated) (%)	755,735.4 (59.3)	620,292.0 (58.0)	135,443.5 (65.8)	<0.001
Hypothyroidism (%)	88,877.9 (7.0)	66,161.2 (6.2)	22,716.6 (11.0)	<0.001
Liver disease (%)	23,594.0 (1.9)	19,614.2 (1.8)	3,979.7 (1.9)	0.176
Neurological disorders (%)	77,865.1 (6.1)	62,038.3 (5.8)	15,826.8 (7.7)	<0.001
Paralysis (%)	25,741.1 (2.0)	20,892.6 (2.0)	4,848.5 (2.4)	<0.001
Obesity (%)	89,688.4 (7.0)	73,405.2 (6.9)	16,283.2 (7.9)	<0.001
Peripheral vascular disorders (%)	79,844.3 (6.3)	57,921.1 (5.4)	21,923.3 (10.6)	<0.001
Psychoses (%)	21,296.2 (1.7)	18,129.4 (1.7)	3,166.9 (1.5)	0.022
Peptic ulcer disease excluding bleeding (%)	2,494.3 (0.2)	1,961.0 (0.2)	533.3 (0.3)	0.001
Valvular disease (%)	55,297.3 (4.3)	34,864.3 (3.3)	20,433.0 (9.9)	<0.001
Weight loss (%)	78,387.5 (6.1)	63,248.7 (5.9)	15,138.9 (7.3)	<0.001

SD, standard deviation; IQR, inter-quartile range; LOS, length of stay.

### Main endpoints

The main endpoints included the temporal trend of AF prevalence in PC inpatients and in-hospital mortality, total cost, length of hospital stay (LOS), complications including congestive heart failure, pulmonary circulation disorders, renal failure, fluid and electrolyte disorders and cardiogenic shock. The total cost was derived from the total charges in the database using the cost-to-charge ratio and Consumer Price Index.

### Statistical analysis

Weighting and stratification methods were used to obtain the total national estimates because the NIS database is based on a sampling design. We then summarized the baseline characteristics of PC patients in the two groups (with or without AF) to demonstrate the descriptive analyses results. We used the independent samples *t*-test or rank sum test and chi-square test to compare the different characteristics between PC patients with and without AF. Mean and standard deviation (SD) were calculated for continuous variables, and frequencies and percentages were calculated for categorical variables.

The Cochran-Armitage trend test was conducted to analyze the temporal trend in AF prevalence, and subgroup analyses were conducted to analyze the temporal trend in AF prevalence based on patient- and hospital-level characteristics. In addition, multivariable-adjusted logistic analyses were conducted to evaluate the association between AF and adverse outcomes (in-hospital mortality, congestive heart failure, pulmonary circulation disorders, renal failure, fluid and electrolyte disorders and cardiogenic shock), controlling for the potential confounders of patient-level, hospital-level, and comorbid factors. The trend in in-hospital mortality was compared between PC inpatients with and without AF. As the total cost and LOS indicated a right-skewed distribution, a logarithmic transformation for these two outcomes was performed before constructing the multivariable linear models.

Sensitivity analyses were performed using the propensity score weighting (PSW) method, which combines logistic regression and overlap weighting (logistic-OW) to balance the variables and assess the outcomes. Absolute standardized mean differences (ASMD) calculated for each covariate between the two groups were used for balance assessment, indicating adequate covariate balance with ASMD < 0.1 (13).

The R software (Version 4.0.3) and SAS (Version 9.4) were used to conduct all the statistical analyses. All tests were two-tailed, and *P* < .05 was considered significant.

### Missing variables

The missing rate for most variables was less than 3%, except for race (9.60%), admission season (4.32%) and median household income of residents in the patient' s ZIP Code (3.15%). We imputed missing data with the dominant category for categorical variables and the median for continuous variables (14). Missing race data were imputed into missing subgroups.

## Results

### Descriptive overview

Among the 256,239 hospitalizations (1,275,345 weighted admissions) aged ≥18 years who were diagnosed with PC from 2008 to 2017 and met the inclusion criteria, 1,069,372 weighted hospitalizations (83.8%) did not have AF, and 205,973 (16.2%) had AF. Comparison of the demographics, hospital characteristics, complications, treatments and clinical outcomes between patients with and without AF is presented in Table 1. For patient-level features, the AF group was characterized by an older age (68.8% vs. 37.0%, *P* < .001), higher proportion of white individuals (74.3% vs. 61.2%, *P* < .001), and greater prevalence of Medicare insurance coverage (84.4% vs. 62.1%, *P *< .001) (Table 1). Regarding comorbidities, the AF group had higher rates of collagen vascular diseases, deficiency anemias, chronic pulmonary disease, coagulopathy, diabetes, hypertension, hypothyroidism, neurological disorders, paralysis, obesity, peripheral vascular disorder, valvular disease, and weight loss. However, these patients were less likely to have lymphatic metastasis or hepatic metastases (Table 1).

### Trends of AF prevalence in PC inpatients and subgroups

**Figure 2:**
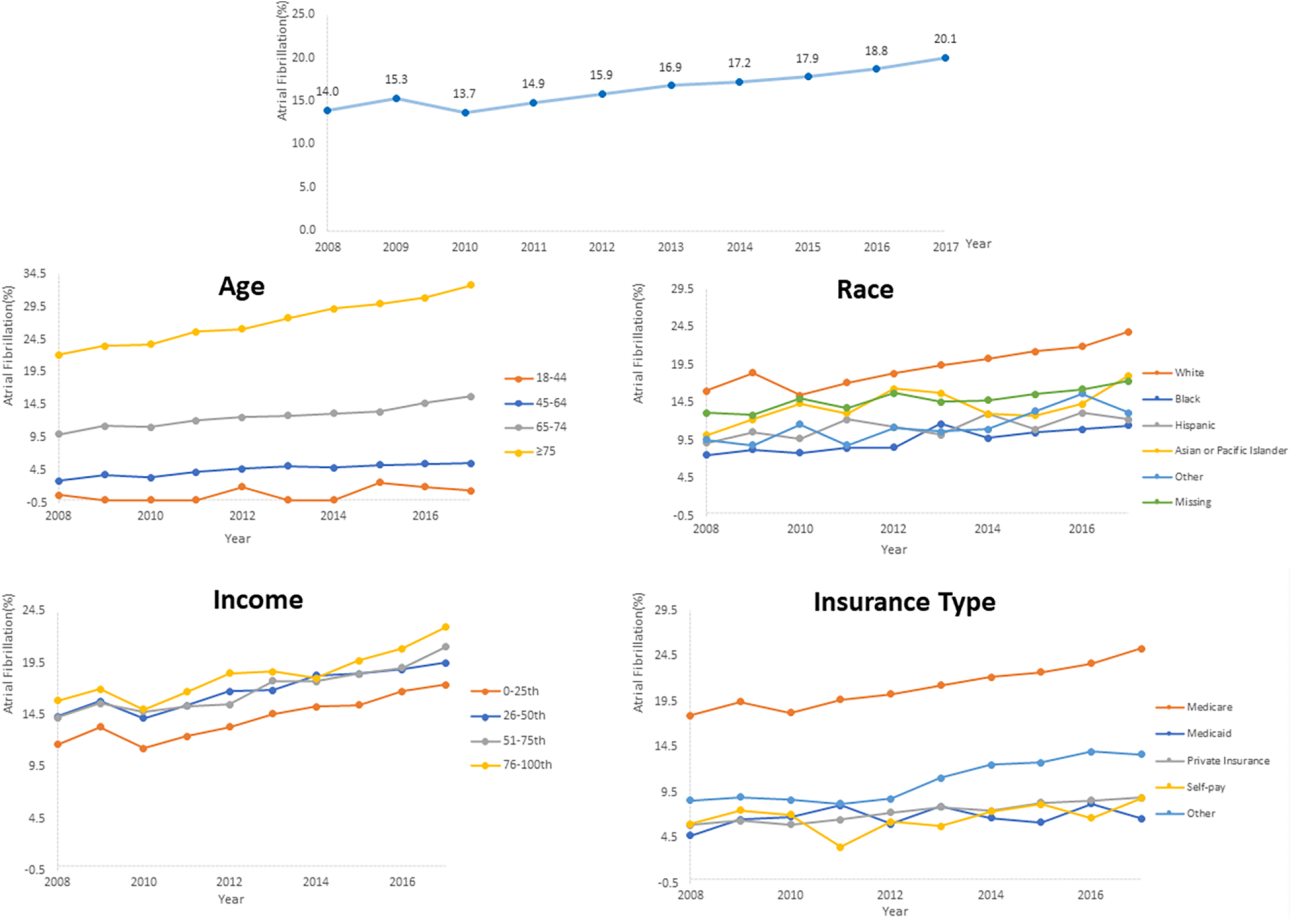
Temporal trends of AF prevalence in PC inpatients subgroups categorized by age, race, insurance and median household income of residents in the patient's ZIP code.

AF prevalence among PC inpatients significantly increased from 14.0% in 2008 to 20.1% in 2017 (*P* for trend <.001). Further subgroup analysis also revealed a consistent rise in AF prevalence across all groups, with all trends being statistically significant (*P* for trend <.001) (Figure 2, Supplementary Figure S1). The prevalence of AF positively correlated with age, with the highest rate observed among patients aged ≥75 years in 2017 (32.8%). Besides, there was an increase in AF prevalence, by 1.9 times, among patients aged 45–64. Furthermore, AF was more prevalent among hospitalized White patients than other racial groups, and the highest prevalence was observed in patients covered by Medicare. Additionally, higher median household income of residents in the patient's ZIP Code and comorbid hypertension were associated with a higher AF prevalence. Supplementary Figure S1 provides further evidence of the consistent increase in AF prevalence across different hospital regions, bed sizes, teaching statuses, and metastatic status reinforcing the findings of this study.

### Association of AF with in-hospital mortality and other clinical outcomes

**Figure 3:**
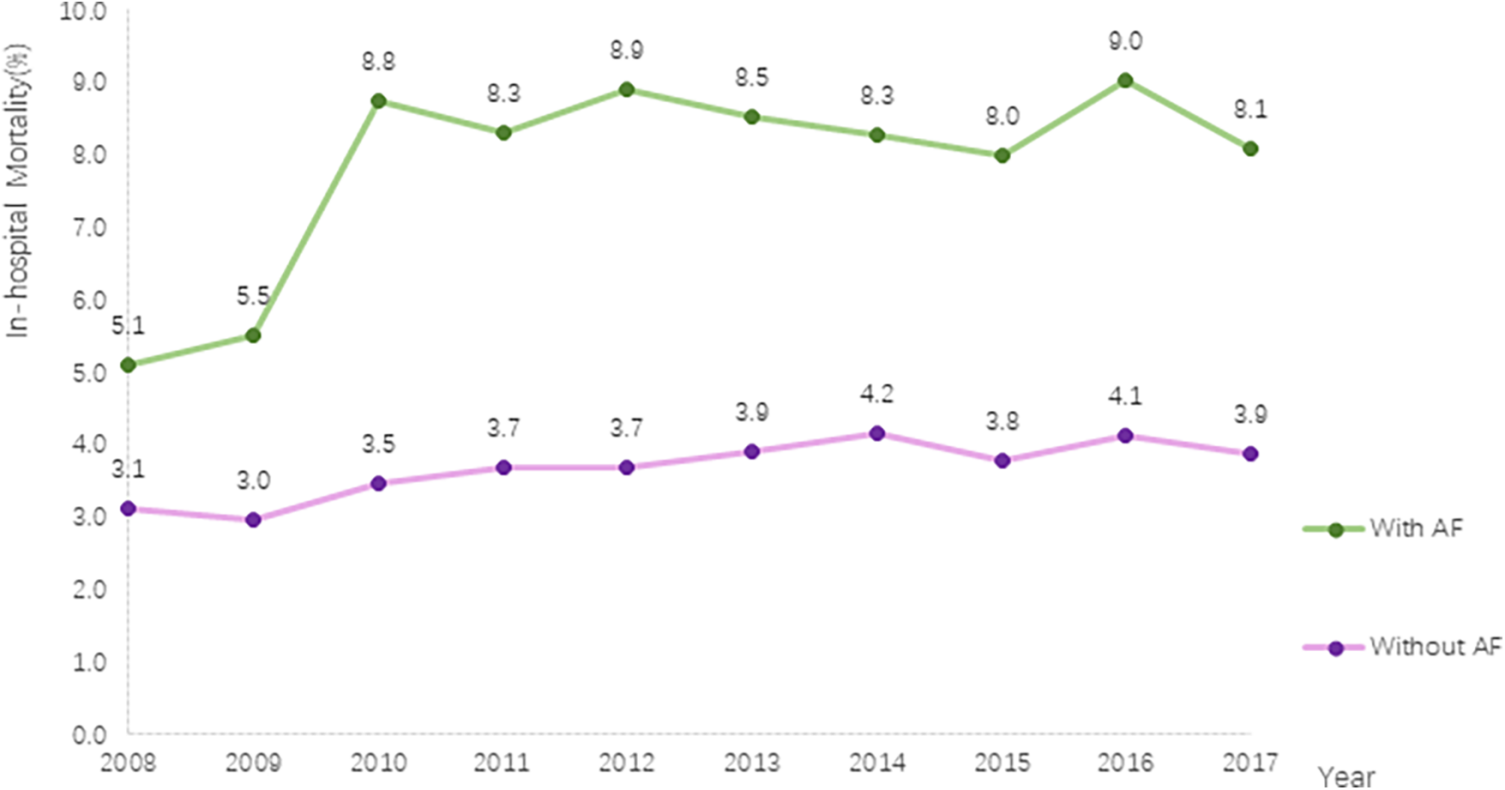
Trends in in-hospital mortality in PC with and without AF.

This study revealed a significant increase in in-hospital mortality among PC inpatients. In patients without AF, the mortality rate increased from 3.1% in 2008 to 3.9% in 2017 (*P* for trend <.001), whereas in patients with AF, the rate increased from 5.1% in 2008 to 8.1% in 2017 (*P* for trend <.001). Throughout the entire study period, patients with AF had a higher in-hospital mortality rate than those without AF (Figure 3).

**Figure 4:**
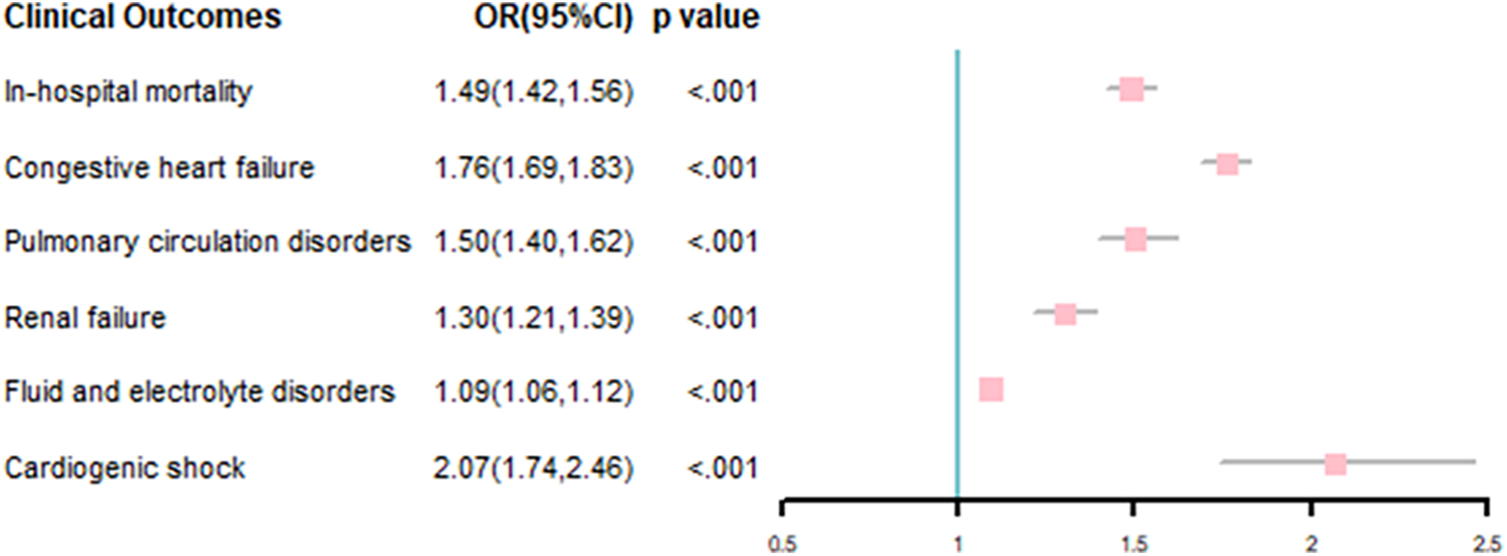
Association of comorbid AF with clinical outcomes in PC inpatients.

Multivariable-adjusted logistic model results showed that AF was associated with incremental in-hospital mortality (odds ration [OR], 1.49; 95% confidence interval [CI], 1.42–1.56), *P *< .001). AF was also associated with other adverse outcomes, including congestive heart failure (OR, 1.76; 95% CI, 1.69–1.83), *P *< .001), pulmonary circulation disorders (OR, 1.50; 95% CI, 1.40–1.62), *P *< .001), renal failure (OR, 1.30; 95% CI, 1.21–1.39), *P *< .001), fluid and electrolyte disorders (OR, 1.09; 95% CI, 1.06–1.12), *P *< .001) and cardiogenic shock (OR, 2.07; 95% CI, 1.74–2.46), *P *< .001; Figure 4).

The subgroup analysis results of patients admitted for radical prostatectomy, PC patients with or without metastatic, and those with or without comorbid hypertension were consistent with the findings of the entire cohort (Supplementary Tables S2,S3).

### *Ad hoc* analysis regarding the insurance type

The relationship between insurance type and AF prevalence, and the association between insurance type and in-hospital mortality were further investigated. Compared with other insurance, even after adjusting covariates, Medicare is still significantly associated with increment of AF prevalence. However, compared with other insurance, patients with Medicare do not undergo an increased risk of death (Supplementary Table S4).

### Association of AF with hospitalization cost and LOS in patients with PC

We also analyzed the median in-hospital LOS and total costs for patients with and without AF.

The median length of stay for patients with AF and without AF were 4.00 days [interquartile range (IQR): 3.00–7.00] and 3.00 days (IQR: 2.00–6.00), respectively. The median total cost for patients with AF was 10,563.17 dollars (IQR: 6,335.66–18,596.05), whereas that for those without AF was 11,045.36 dollars (IQR: 6,784.84–17,179.64) (Table 1). After performing a logarithmic transformation and multivariate regression, we found that the AF group had a 9% higher total cost and 9% longer LOS. Furthermore, the LOS and total costs for inpatients with and without AF decreased slightly over the entire study period (Table 2).

**Table 2:** Association of AF With total cost and LOS in patients with PC from 2008 to 2017.

Items	Estimates	95%CI	*P* value
Association of AF compared with no AF with each outcome among all participants			
Total cost	0.09	(0.08, 0.10)	<.001
LOS	0.09	(0.08, 0.09)	<.001
Association of each outcome with unit increase in year for inpatients with AF			
Total cost	−0.03	(−0.03, −0.02)	<.001
LOS	−0.02	(−0.02, −0.01)	<.001
Association of each outcome with unit increase in year for inpatients without AF			
Total cost	−0.01	(−0.01, −0.01)	<.001
LOS	−0.01	V−0.01, −0.01)	<.001

Obtained from the general linear model with either cost or LOS as the dependent variable and AF as the independent variable, adjusted for age, race, median household income of residents in the patient’ s ZIP Code, insurance type, hospital region, hospital location/teaching status, hospital bed size, hospital ownership, discharge year, and comorbidities after accounting for survey design. AF, atrial fibrillation; PC, prostate cancer; LOS, length of stay.

### Sensitivity analyses

Most of the baseline variables were unbalanced between the AF and without AF groups. After PSW model, all the ASMD were <.001, suggesting a good balance between these comparisons (Supplementary Figure S2). In the PSW, we obtained similar results, showing that AF group was associated with incremental in-hospital mortality (OR, 1.40; 95% CI, 1.34–1.46, *P *< .001), congestive heart failure (OR, 1.57; 95% CI, 1.52–1.62, *P *< .001), pulmonary circulation disorders (OR, 1.47; 95% CI, 1.37–1.57, *P *< .001), renal failure (OR, 1.04; 95% CI, 1.02–1.07, *P *< .001), fluid and electrolyte disorders (OR, 1.08; 95% CI, 1.06–1.11, *P *< .001) and cardiogenic shock (OR, 1.81; 95% CI, 1.54–2.14, *P *< .001) (Supplementary Table S5). In addition, AF group had a higher total cost and longer LOS than the without AF group (Supplementary Table S5).

## Discussion

This contemporary analysis is the largest nationally representative study focusing on AF prevalence in a PC population. We observed increased AF prevalence among PC inpatients between 2008 and 2017. Meanwhile, a significantly higher AF prevalence was observed, particularly in the aging, White, high median household income of residents in the patient's ZIP Code, and Medicare-receiving populations. In-hospital mortality rate in PC patients with AF rapidly increased over the study period, and AF was associated with higher in-hospital mortality and healthcare resource utilization, including higher costs and longer LOS. Sensitivity analyses using different statistical models ensured the robustness and reliability of these results. Considering the heavy health burden caused by both AF and PC, our study highlighted their current status and raised awareness regarding the necessity for improved AF management and control.

AF, caused by abnormal electrical activity within the atria of the heart, is the most common clinical arrhythmia and results in adverse outcomes and increased healthcare costs (15). AF etiology may be related to an imbalance between the sympathetic and the parasympathetic system activities, which is also observed in certain cancer patients (16). In advance, paraneoplastic syndromes, inflammation, abnormal homeostasis, and the consumptive symptoms including anemia caused by malignancy aggravate the risks of AF, collectively or respectively (17). Moreover, cancer treatments, including chemotherapy and radiation therapy, may lead to structural damage that increases AF risk.

Patients with AF are markedly predisposed to platelet dysregulation. Notably, there exists a significant bidirectional interaction between cancer cells and platelets: oncological processes modulate platelet physiology; in turn, activated platelets contribute to every stage of cancer progression, facilitating tumor expansion, angiogenesis, metastasis, and the emergence of cancer-associated thrombosis (17). Consequently, patients with cancer who develop AF are at substantially higher risk of ischemic events without anti-thrombotic treatment or hemorrhage with anti-thrombotic treatment compared with those suffering only AF (18). With the prolonged survival of cancer patients, cancer is an important risk factors for cardiovascular disease and is gradually being taken into clinical consideration (19).

PC is the most frequently diagnosed cancer among males and has the third leading cause of cancer-related deaths in males in developed countries (20). The incidence of PC tends to increase with age and may present within a population with an increased risk of CVD comorbidities (21). PC accounts for one in five male cancer diagnoses, especially in those who receive hormonal therapy. Testosterone in males and progesterone in females have corrected QT interval shortening effects (3, 22). Androgen deprivation therapy (ADT) exposes males to considerable cardiovascular risks, including important electrophysiological changes (23). Meanwhile, electrophysiological changes, including AF, have been reported in those receiving cancer treatments using exogenous hormonal drugs (24). Prolonged ADT exposure in an aging populations is associated with increased cardiovascular morbidity (25). In recent years, the progressive widespread use of novel antiandrogen therapies such as abiraterone and enzalutamide, has also targeted the hormonal axis. This application has led to further clinical concerns regarding the possible cardiovascular mortality risk in patients (26).

Regardless of hormonal therapy, radical prostatectomy is the most commonly used treatment modality for the disease, and still remains the reference standard treatment for localized PC (27). As a common postoperative complication, perioperative AF is highly associated with adverse outcomes such as cardiac complications, longer hospital stays, higher costs, and higher resource utilization (12, 28).

Previous population-based studies reported that age and race are independent risk factors for AF. In our study, AF incidence increased to varying degrees in different subgroups of patients during the study period. Consistent with the results of previous studies, the highest incidence rates were observed in advanced age and the White race groups (29). The proportion of patients with advanced age increased as the life expectancy of PC patients has increased; however, the increased incidence of comorbidities owing to the increased life expectancy has not received sufficient attention. The presence of a history of cancer may lead to overlooking the risk of comorbidities such as AF in clinical practice, resulting in treatment delays.

In the model adjusted for sociodemographic factors, health insurance, income level, perceived stress, cardiovascular risk factors, and unemployment were associated with a 60% increase in the odds for AF. This association was consistent in subgroups stratified by median age, sex, race, education, income, and health insurance status (30). In our study, AF prevalence was higher in high median household income of residents in the patient's ZIP Code group than in low income groups, which conflicts with the results of the aforementioned studies. Considering these results and AF prevalence in patients with different health insurance types, the reason may be related to the relatively underdeveloped medical care in low-income areas and limitations of the patients' ability to pay, resulting in a lower rate visits and detection of AF. Additionally, a patients' cancer history may further reduce their ability and willingness to receive treatment, possibly leading to underdiagnosis and active abandonment of relevant treatment. Furthermore, in our study, we found that compared with other insurance type AF prevalence was higher among individuals covered by Medicare. However, being supported by Medicare was not significantly correlated with increased in-hospital mortality. These findings indicate the healthcare reimbursed by Medicare for PC patients who comorbid with AF is sufficient. A specialized study for relevant populations is anticipated in the future to further analyze different populations.

This study had some limitations. Firstly, we could not exclude potential inaccuracies owing to coding errors. All codes used were verified in previous studies, and such coding errors were likely to be random rather than systemic because of the large size of the NIS database. Secondly, owing to the nature of the NIS database design, we could not distinguish index admissions from readmitted admissions nor can we analyze the number of hospitalizations per patient, which may have resulted in an overestimation of AF prevalence among individual patients. Although we could not determine the exact prevalence of AF in individuals, considering the large sample size and significant increase in AF prevalence in hospital encounters in this study, we assumed that the prevalence of AF in individuals with PC has also increased between 2008 and 2017. Thirdly, the database used in our study did not include treatment-related information. Therefore, future studies could incorporate additional data sources or registries that provide comprehensive treatment details to better understand the impact of various PC treatments on AF development and outcomes.

Our findings statistically and epidemiologically uncovered the association between PC and AF prevalence, as well as their significant adverse clinical outcomes. More ever, we are carrying out advanced research into the biological or mechanistic links between these conditions to offer valuable insights into causality and potential intervention points. No doubt, including cost-effectiveness analyses of AF management strategies in PC patients could help in understanding the economic implications and guiding healthcare policy decisions.

## Conclusions

Our study revealed the current temporal trends and characteristics of AF among hospital encounters with PC in the United States. AF incidence in PC patients has increased in the past decade. AF was associated with poor prognosis and high health resource utilization. Better management strategies for PC patients with AF comorbid, especially older individuals, are necessary. To avoid the adverse consequences of missed diagnoses, more attention should be paid to comorbidities in clinical practice.

## Data Availability

Publicly available datasets were analyzed in this study. This data can be found here: https://hcup-us.ahrq.gov/.
