# Supplementary material for: Prevalence and outcomes of atrial fibrillation in patients suffering prostate cancer: a national analysis in the United States
Source: Front Cardiovasc Med. 2024 Apr 4;11:1382166. doi: 10.3389/fcvm.2024.1382166 (PMC11025351; doi:10.3389/fcvm.2024.1382166)
Supplement: Supplementary file 1 [file Table1.docx]

**SUPPLEMENTARY TABLE 1The ICD Codes**

| **Item** | **ICD-9** | **ICD-9 NAME** | **ICD-10** | **ICD-10 NAME** |
| --- | --- | --- | --- | --- |
| Prostate cancer | 233.4 | Carcinoma in situ of prostate | D07.5 | Carcinoma in situ of prostate |
|  | 185 | Malignant neoplasm of prostate | C61 | Malignant neoplasm of prostate |
| Atrial fibrillation | 427.3 | Atrial fibrillation and flutter | I48 | Atrial fibrillation and flutter |
| Chemotherapy | 992.5 | Injection or infusion of cancer chemotherapeutic substance | 3E0.3305 | Introduction of Other Antineoplastic into Peripheral Vein, Percutaneous Approach |
|  |  |  | 3E0.4305 | Introduction of Other Antineoplastic into Central Vein, Percutaneous Approach |
|  |  |  | XW0.3351 | Introduction of Blinatumomab Antineoplastic Immunotherapy into Peripheral Vein, Percutaneous Approach, New Technology Group 1 |
|  |  |  | XW0.4351 | Introduction of Blinatumomab Antineoplastic Immunotherapy into Central Vein, Percutaneous Approach, New Technology Group 1 |
| Radiotherapy | 922 | Therapeutic radiology and nuclear medicine | D | Radiation Therapy |
| Lymph node metastasis | 196.5 | Secondary and unspecified malignant neoplasm of lymph nodes of inguinal region and lower limb | C77.4 | Secondary and unspecified malignant neoplasm of inguinal and lower limb lymph nodes |
|  | 196.6 | Secondary and unspecified malignant neoplasm of intrapelvic lymph nodes | C77.5 | Secondary and unspecified malignant neoplasm of intrapelvic lymph nodes |
| Brain metastases | 198.3 | Secondary malignant neoplasm of brain and spinal cord | C79.31 | Secondary malignant neoplasm of brain |
| Bone metastases | 198.5 | Secondary malignant neoplasm of bone and bone marrow | C79.51 | Secondary malignant neoplasm of bone |
|  |  |  | C79.52 | Secondary malignant neoplasm of bone marrow |
| Pulmonary metastasis | 197.0 | Secondary malignant neoplasm of lung | C78.00 | Secondary malignant neoplasm of unspecified lung |
| Hepatic metastases | 197.7 | Malignant neoplasm of liver, secondary | C78.7 | Secondary malignant neoplasm of liver and intrahepatic bile duct |
| Tobacco abuse | 649.0 | Tobacco use disorder complicating pregnancy, childbirth, or the puerperium | O99.33 | Tobacco use disorder complicating pregnancy, childbirth, and the puerperium |
|  | 305.1 | Tobacco use disorder | F17 | Nicotine dependence |
| Smoking history | V15.82 | Personal history of tobacco use | Z87.891 | Personal history of nicotine dependence |
| CHD | 410 | Acute myocardial infarction | I20 | Angina pectoris |
|  | 411 | Other acute and subacute forms of ischemic heart disease | I21 | Acute myocardial infarction |
|  | 412 | Old myocardial infarction | I22 | Subsequent myocardial infarction |
|  | 413 | Angina pectoris | I23 | Certain current complications following acute myocardial infarction |
|  | 414 | Other forms of chronic ischemic heart disease | I24 | Other acute ischaemic heart diseases |
|  |  |  | I25 | Chronic ischaemic heart disease |
| Lipoid disorder | 272 | Disorders of lipoid metabolism | E78 | Disorders of lipoprotein metabolism and other lipidaemias |
| Atherosclerosis | 440 | Atherosclerosis | I70 | Other disorders of arteries and arterioles |
|  | 414.0 | Coronary atherosclerosis | I25.1 | Atherosclerotic heart disease of native coronary artery |
|  |  |  | I25.81 | Atherosclerosis of other coronary vessels without angina pectoris |
|  | 414.3 | Coronary atherosclerosis due to lipid rich plaque | I25.83 | Coronary atherosclerosis due to lipid rich plaque |
|  | 414.4 | Coronary atherosclerosis due to calcified coronary lesion | I25.84 | Coronary atherosclerosis due to calcified coronary lesion |
|  |  |  | I25.7 | Atherosclerosis of coronary artery bypass graft(s) and coronary artery of transplanted heart with angina pectoris |
|  | 437.0 | Cerebral atherosclerosis | I67.2 | Cerebral atherosclerosis |
| Nephrotic syndrome | 581 | Nephrotic syndrome | N04 | Nephrotic syndrome |
| Hyperthyroidism | 242 | Thyrotoxicosis with or without goiter | E50 | Thyrotoxicosis [hyperthyroidism] |
| Chronic kidney disease | 585 | Chronic kidney disease (ckd) | N18 | Chronic kidney disease (CKD) |
| Cardiogenic shock | 785.51 | Cardiogenic shock | R57.0 | Cardiogenic shock |
| Metastatic | 198.5 | bone and bone marrow | C79.51  C79.52 | Secondary malignant neoplasm of bone Secondary malignant neoplasm of bone marrow |
|  | 196 | distant lymph nodes | C77 | Secondary and unspecified malignant neoplasm of lymph nodes |
|  | 197.7 | liver | C78.7 | Secondary malignant neoplasm of liver and intrahepatic bile duct |
|  | 197.0 197.2 197.1 197.3 | thorax | C78.0 C78.2 C78.1 C78.3 | Secondary malignant neoplasm of lung Secondary malignant neoplasm of pleura Secondary malignant neoplasm of mediastinum Secondary malignant neoplasm of other and unspecified respiratory organs |
|  | 198.7 198.0 | adrenal gland and kidney | C79.7 C79.0 | Secondary malignant neoplasm of adrenal gland Secondary malignant neoplasm of kidney and renal pelvis |
|  | 198.3 | brain and spinal cord | C79.31 | Secondary malignant neoplasm of brain |
|  | 197.6 | retroperitoneum and peritoneum | C78.6 | Secondary malignant neoplasm of retroperitoneum and peritoneum |
|  | 197.5 197.4 197.8 | digestive system | C78.5 C78.4 C78.7 C78.89 | Secondary malignant neoplasm of large intestine and rectum Secondary malignant neoplasm of small intestine Secondary malignant neoplasm of liver and intrahepatic bile duct Secondary malignant neoplasm of other digestive organs |
