# Supplementary material for: Prevalence and outcomes of atrial fibrillation in patients suffering prostate cancer: a national analysis in the United States
Source: Front Cardiovasc Med. 2024 Apr 4;11:1382166. doi: 10.3389/fcvm.2024.1382166 (PMC11025351; doi:10.3389/fcvm.2024.1382166)
Supplement: Supplementary file 2 [file Table2.docx]

| **SUPPLEMENTARY TABLE 2 Association of comorbid AF with clinical outcomes in PC inpatients admitted for radical prostatectomy operations** | | | |
| --- | --- | --- | --- |
| **Items** | **Without AF** | **With AF** | |
|  | **Ref** | **OR (95%CI)** | ***P*-value** |
| In-hospital mortality | Ref | 3.34(1.61,6.91) | 0.0012 |
| Congestive heart failure | Ref | 5.18(4.00,6.70) | <.0001 |
| Pulmonary circulation disorders | Ref | 3.33(2.18,5.09) | <.0001 |
| Renal failure | Ref | 1.10(0.81,1.50) | 0.5485 |
| Fluid and electrolyte disorders | Ref | 1.71(1.46,1.99) | <.0001 |
| Cardiogenic shock | Ref | 5.22(1.66,16.42) | 0.0047 |
| AF: atrial fibrillation; PC: prostate cancer; OR: odds ratio; CI: confidence interval. | | | |
