# Supplementary material for: Prevalence and outcomes of atrial fibrillation in patients suffering prostate cancer: a national analysis in the United States
Source: Front Cardiovasc Med. 2024 Apr 4;11:1382166. doi: 10.3389/fcvm.2024.1382166 (PMC11025351; doi:10.3389/fcvm.2024.1382166)
Supplement: Supplementary file 3 [file Table3.docx]

**SUPPLEMENTARY TABLE 3 Association of comorbid AF with in-hospital mortality among different subgroups of PC inpatients**

| **Items** | **Without AF** | **With AF** | |
| --- | --- | --- | --- |
|  | **Ref** | **OR (95%CI)** | ***P*-value** |
| - Metastatic |  |  |  |
| In-hospital mortality | Ref | 1.44(1.32,1.57) | <.0001 |
| - Non-metastatic |  |  |  |
| In-hospital mortality | Ref | 1.55(1.46,1.64) | <.0001 |
| - With Hypertension |  |  |  |
| In-hospital mortality | Ref | 1.54(1.45,1.64) | <.0001 |
| - Without Hypertension |  |  |  |
| In-hospital mortality | Ref | 1.41(1.31,1.52) | <.0001 |
| AF: atrial fibrillation; PC: prostate cancer; OR: odds ratio; CI: confidence interval. | | | |
