# Supplementary material for: Prevalence and outcomes of atrial fibrillation in patients suffering prostate cancer: a national analysis in the United States
Source: Front Cardiovasc Med. 2024 Apr 4;11:1382166. doi: 10.3389/fcvm.2024.1382166 (PMC11025351; doi:10.3389/fcvm.2024.1382166)
Supplement: Supplementary file 4 [file Table4.docx]

**SUPPLEMENTARY TABLE 4 Association of insurance type with AF prevalence and in-hospital mortality**

|  | **Medicare** | **Medicaid** | **Private ins** | **Self-pay** | **Other** |
| --- | --- | --- | --- | --- | --- |
| **Association of insurance type with AF prevalence** | | | | | |
| - all age group | | | | | |
| OR (95%CI) | Ref | 0.84(0.77,0.93) | 0.86(0.83,0.90) | 0.81(0.69,0.94) | 0.91(0.83,0.99) |
| P-value |  | 0.0003 | <.0001 | 0.0056 | 0.0346 |
| - ≥65 years | | | | | |
| OR (95%CI) | Ref | 0.83(0.73,0.95) | 0.89(0.85,0.94) | 0.81(0.68,0.98) | 0.92(0.83,1.02) |
| P-value |  | 0.0063 | <.0001 | 0.0308 | 0.1164 |
| **Association of insurance type with In-hospital mortality** | | | | | |
| OR (95%CI) | Ref | 1.17(0.85,1.62) | 1.32(1.15,1.52) | 1.36(0.81,2.29) | 2.01(1.55,2.60) |
| P-value |  | 0.3249 | 0.0001 | 0.2443 | <.0001 |

Obtained from the logistic regression model with if combine with AF or in-hospital mortality as the dependent variable and insurance type as the independent variable, adjusted for age, race, median household income of residents in the patient’ s ZIP Code, hospital region, hospital location/teaching status, hospital bed size, hospital ownership, discharge year, and comorbidities after accounting for survey design.
