# Supplementary material for: Prevalence and outcomes of atrial fibrillation in patients suffering prostate cancer: a national analysis in the United States
Source: Front Cardiovasc Med. 2024 Apr 4;11:1382166. doi: 10.3389/fcvm.2024.1382166 (PMC11025351; doi:10.3389/fcvm.2024.1382166)
Supplement: Supplementary file 5 [file Table5.docx]

| **SLUPPLEMENTARY TABLE 5 Sensitivity Analysis** | | |  |
| --- | --- | --- | --- |
| **Items** | **Without AF** | **With AF** | |
|  | **Ref** | **OR (95%CI)** | ***P*-value** |
| In-hospital mortality | Ref | 1.40(1.34,1.46) | <.001 |
| Congestive heart failure | Ref | 1.57(1.52,1.62) | <.001 |
| Pulmonary circulation disorders | Ref | 1.47(1.37,1.57) | <.001 |
| Renal failure | Ref | 1.04(1.02,1.07) | 0.001 |
| Fluid and electrolyte disorders | Ref | 1.08(1.06,1.11) | <.001 |
| Cardiogenic shock | Ref | 1.81(1.54,2.14) | <.001 |
|  | **Ref** | **Estimate(95%CI)** | ***P*-value** |
| LOS | Ref | 0.09(0.09,0.10) | <.001 |
| Total Cost | Ref | 0.09(0.08,0.10) | <.001 |
| Note: Propensity score weighting analysis of clinical outcomes in patients with PC; | | | |
| AF: atrial fibrillation; PC: prostate cancer; OR: odds ratio; CI: confidence interval. | | | |
