# Supplementary figures and images for: Prevalence and outcomes of atrial fibrillation in patients suffering prostate cancer: a national analysis in the United States
Source: Front Cardiovasc Med. 2024 Apr 4;11:1382166. doi: 10.3389/fcvm.2024.1382166 (PMC11025351; doi:10.3389/fcvm.2024.1382166)

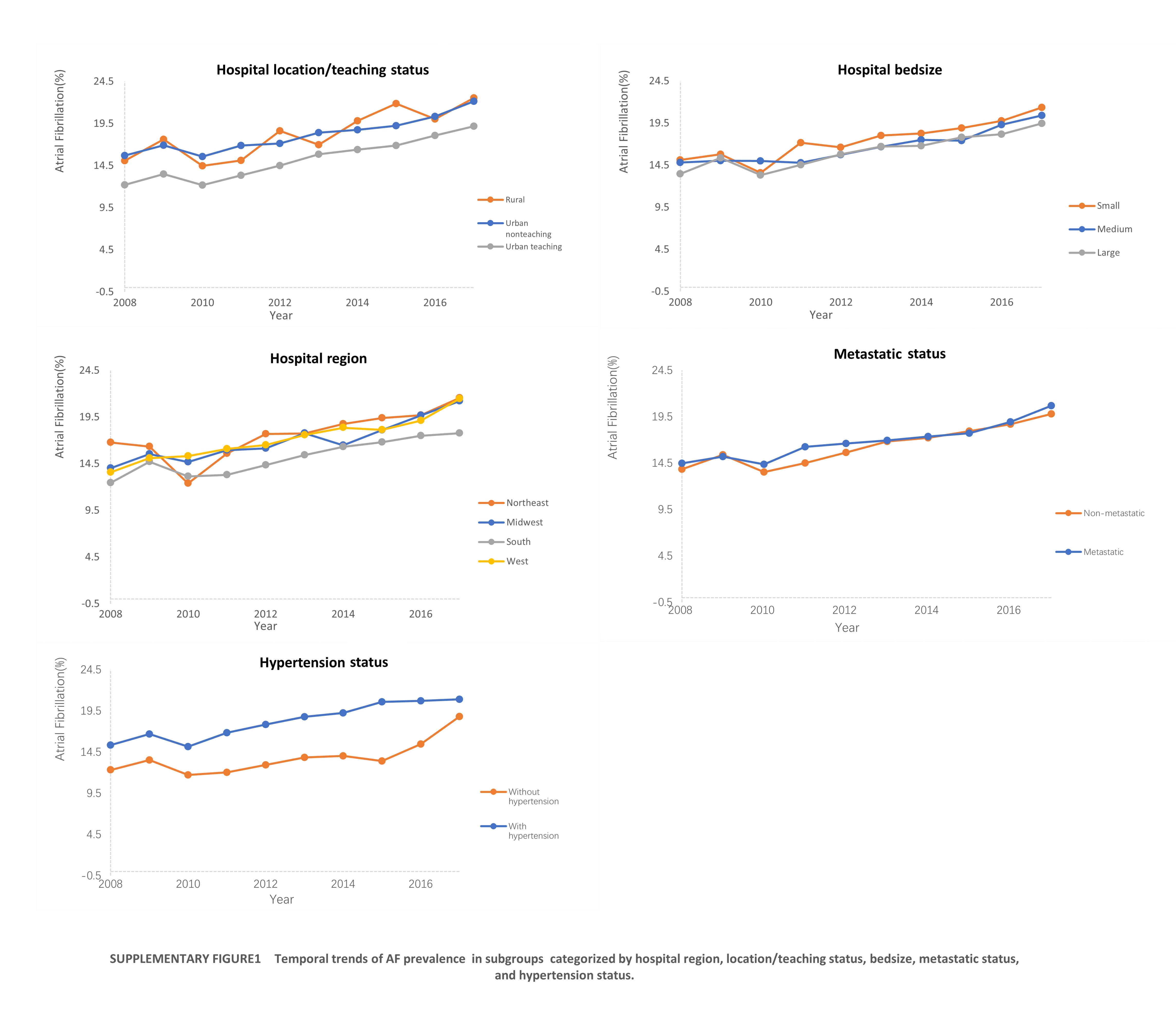

Supplement: Supplementary file 6 [file Image1.tif]

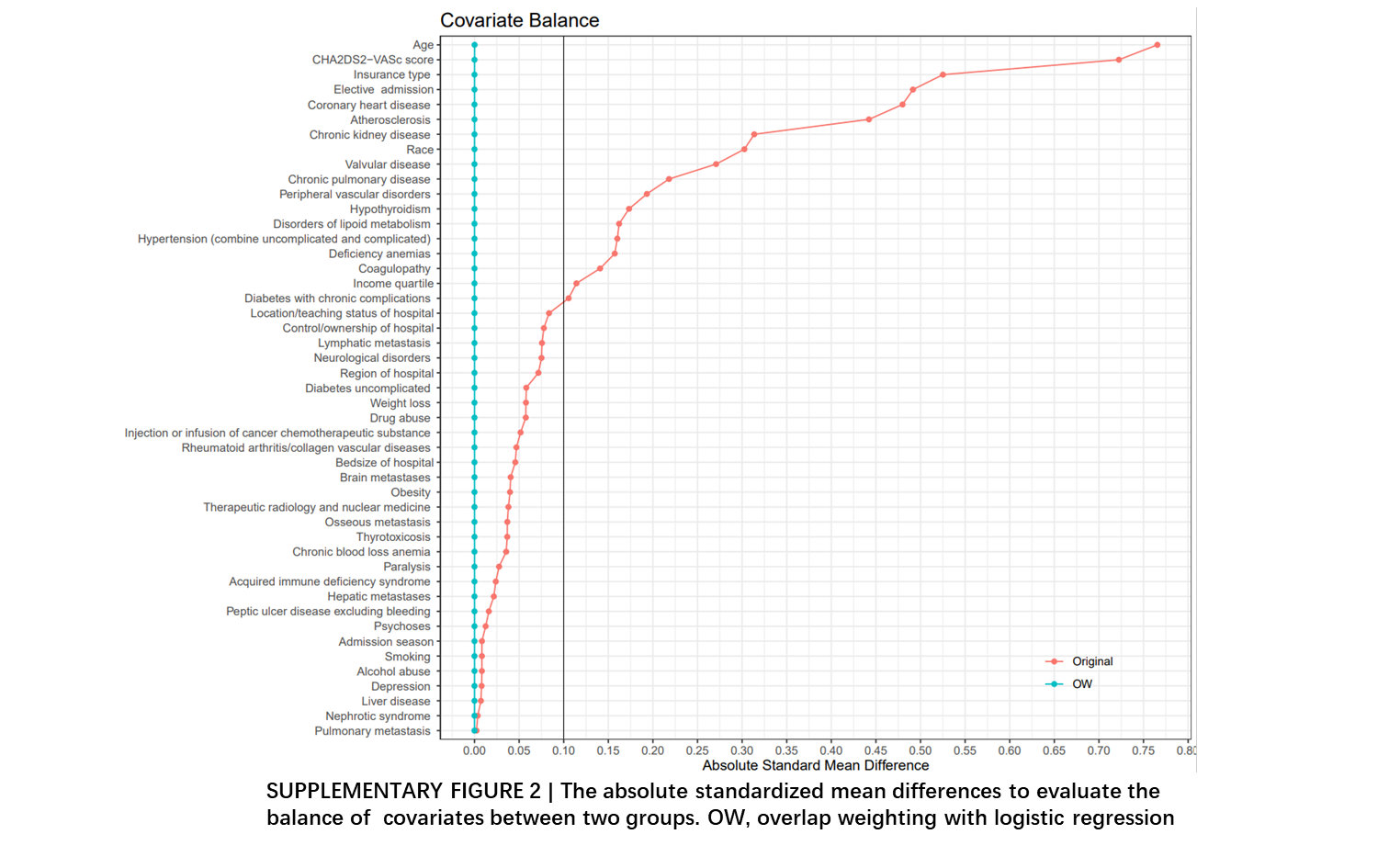

Supplement: Supplementary file 7 [file Image2.tif]
